# Glucocerebrosidase Deficiency in Substantia Nigra of Parkinson Disease Brains

**DOI:** 10.1002/ana.23614

**Published:** 2012-10-02

**Authors:** Matthew E Gegg, Derek Burke, Simon J R Heales, J Mark Cooper, John Hardy, Nicholas W Wood, Anthony H V Schapira

**Affiliations:** 1 Department of Clinical Neurosciences, University College London Institute of NeurologyLondon, United Kingdom; 2 Molecular and Genetics Unit, University College London Institute of Child HealthLondon, United Kingdom; 3 Enzyme Unit and Metabolic Unit, Chemical Pathology, Great Ormond Street HospitalLondon, United Kingdom; 4 Reta Lila Weston Research Laboratories, Departments of Molecular Neuroscience and Clinical Neuroscience, University College London Institute of NeurologyLondon, United Kingdom; 5 Department of Molecular Neuroscience, University College London Institute of NeurologyLondon, United Kingdom

## Abstract

**Objective:**

Mutations in the glucocerebrosidase gene (*GBA*) represent a significant risk factor for developing Parkinson disease (PD). We investigated the enzymatic activity of glucocerebrosidase (GCase) in PD brains carrying heterozygote *GBA* mutations (PD+GBA) and sporadic PD brains.

**Methods:**

GCase activity was measured using a fluorescent assay in cerebellum, frontal cortex, putamen, amygdala, and substantia nigra of PD+GBA (n = 9–14) and sporadic PD brains (n = 12–14). Protein expression of GCase and other lysosomal proteins was determined by western blotting. The relation between GCase, α-synuclein, and mitochondria function was also investigated in vitro.

**Results:**

A significant decrease in GCase activity was observed in all PD+GBA brain areas except the frontal cortex. The greatest deficiency was in the substantia nigra (58% decrease; *p* < 0.01). GCase activity was also significantly decreased in the substantia nigra (33% decrease; *p* < 0.05) and cerebellum (24% decrease; *p* < 0.05) of sporadic PD brains. GCase protein expression was lower in PD+GBA and PD brains, whereas increased C/EBP homologous protein and binding immunoglobulin protein levels indicated that the unfolded protein response was activated. Elevated α-synuclein levels or PTEN-induced putative kinase 1 deficiency in cultured cells had a significant effect on GCase protein levels.

**Interpretation:**

GCase deficiency in PD brains with *GBA* mutations is a combination of decreased catalytic activity and reduced protein levels. This is most pronounced in the substantia nigra. Biochemical changes involved in PD pathogenesis affect wild-type GCase protein expression in vitro, and these could be contributing factors to the GCase deficiency observed in sporadic PD brains. ANN NEUROL 2012;72:455–463.

The lysosomal storage disorder Gaucher disease (GD) is caused by autosomal recessive mutations in the glucocerebrosidase (*GBA*) gene. *GBA* encodes a lysosomal enzyme (GCase) that catalyses the metabolism of the sphingolipid glucosylceramide to ceramide and glucose. Deficiency of GCase activity results in accumulation of substrate in the lysosomes of several tissues, including brain. Mutations in *GBA* result in 3 clinical manifestations. Type 1 GD occurs in both children and adults and predominantly impacts on the non-neuronal organs, whereas types 2 and 3 have an onset in childhood and adolescence, respectively, and exhibit neurological deficits.^1^

Parkinson disease (PD) is primarily characterized by the motor symptoms of resting tremor, bradykinesia, rigidity, and postural instability. Pathological hallmarks include loss of dopaminergic neurons from the substantia nigra (SN) and the presence of cytoplasmic inclusions known as Lewy bodies in the surviving cells of affected brain regions.^2^

Typical parkinsonism is among the neurological complications of GD (including type 1).^3^, ^4^ The neuropathology of GD brains includes the typical hallmarks of PD, such as cortical and brainstem Lewy bodies.^5^ Heterozygote carriers of *GBA* mutations also have an increased frequency of PD, and these mutations are the most common genetic risk factor for developing the disease.^6^–^8^

Although the pathogenesis of PD is still unknown, the accumulation of α-synuclein and other ubiquitinated proteins in Lewy bodies has implicated protein mishandling as a putative cause. The proteasome and lysosomes are the 2 principal mechanisms for degrading cellular constituents. Autophagy utilizes lysosomes to degrade long-lived proteins, misfolded/aggregated proteins, and organelles such as mitochondria.^9^ Defective autophagy and/or lysosomal depletion have been implicated in PD.^10^–^13^ Cellular or animal models of GCase deficiency have caused α-synuclein accumulation.^14^–^18^ GCase has also been suggested to bind directly with α-synuclein in lysosomes^19^ and the GCase substrate glucosylceramide stabilizes soluble oligomeric α-synuclein species.^18^ These observations have led to the notion that GCase deficiency might contribute to the α-synuclein aggregation characteristic of PD pathology.

Despite the recognized association between *GBA* mutations and PD, it is unknown how heterozygous *GBA* mutations affect GCase activity in PD brains. In this paper, we provide the first report of the activity of GCase in several regions of PD brains from *GBA* mutation carriers and sporadic PD brains. GCase deficiency was greatest in the SN of PD brains with *GBA* mutations. This loss of activity was in part mediated by a decrease in GCase protein levels. GCase activity was also significantly decreased in the SN of sporadic PD brains.

## Materials and Methods

### Brain Samples

Control brains (n = 10), PD brains from *GBA* mutation carriers (PD+GBA; n = 14), and sporadic PD brains (n = 14) were obtained from the Queen Square Brain Bank for Neurological Disorders (London, UK) following local ethical approval. All PD cases met the UK Brain Bank Clinical Criteria for PD. The 3 groups were matched for age (control, 67.7 ± 6.0 years; PD+GBA, 67.5 ± 2.8 years; PD, 68.9 ± 2.8 years) and postmortem delay (control, 53.5 ± 8.1 hours; PD+GBA, 50.5 ± 6.6 hours; PD, 41.8 ± 5.0 hours). GCase enzyme activity was not influenced by postmortem delay (Supplementary Fig 1). Details of *GBA1* mutations are listed in the Table.

**Table 1:** Parkinson Disease Brains with Heterozygote *GBA* Mutations

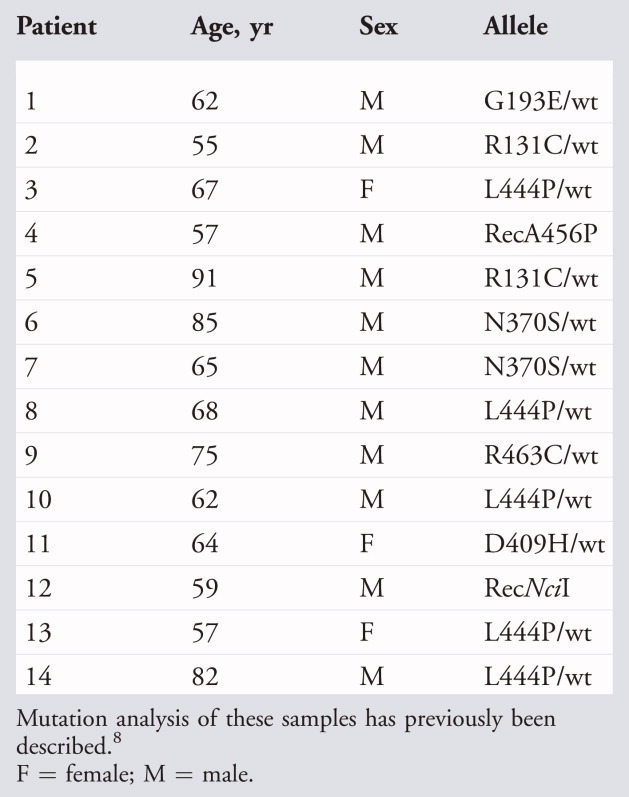

### Enzymatic Assays

Brain samples were homogenized in 250mM sucrose, 10mM Tris (pH 7.4), 1mM ethylenediaminetetraacetic acid supplemented with protease inhibitors (1mM phenylmethanesulfonyl fluoride, 1μg/ml pepstatin A, 1μg/ml leupeptin), and 1mM NaVO_4_. Homogenate was diluted to 2mg/ml in water and sonicated, and GCase activity was determined in samples (20μg protein) by hydrolysis of 5mM 4-methylumbelliferyl-β-D-glucopyranoside in McIIvaine buffer (pH 5.4) in the presence of 22mM sodium taurocholate at 37°C for 1 hour.^20^ The reaction was stopped by addition of 0.25M glycine (pH 10.4) and 4-methylumbelliferone fluorescence measured at excitation 365nm, emission 450nm.

Measurement of nonlysosomal GCase (GBA2) was performed as above but in the absence of sodium taurocholate and with the addition of 1μM deoxynojirimycin, a GBA2 inhibitor.^21^

β-Hexosaminidase was assayed in the above homogenates (2μg protein) using the fluorogenic substrate 4-methylumbelliferyl-2-acetoamido-2-deoxy-6-sulfo-β-D-glucopyransoside (2mM) in sodium citrate buffer (pH 4.2) at 37°C for 30 minutes. The reaction was stopped by addition of 0.25M glycine (pH 10.4), and fluorescence was measured as above.^22^

### Western Blotting

Brain homogenates were mixed 1:1 with 1% (vol/vol) Triton X-100, and debris was removed by centrifugation at 17,000 × *g*. Supernatant (25μg protein) was separated on 4 to 12% or 12% (LC3-II blots) NuPAGE Tris-Bis gels (Invitrogen, Carlsbad, CA), transferred to Hybond P (GE Healthcare, Milwaukee, WI), and probed with primary and respective horseradish peroxidase–conjugated secondary antibodies. Bands were detected by enhanced chemiluminescence (Pierce, Rockford, IL), and density was measured using Image J software (NIH). Protein expression was expressed as a ratio against β-actin.

Details of mammalian cell culture, quantitative polymerase chain reaction, antibodies, GCase RNA interference (RNAi), soluble/insoluble fractionation, endoglycosidase-H (endo-H) assay, lysosomal integral membrane protein (LIMP)-2 immunoprecipitation, Xbp-1 splicing, and statistical analyses can be found in the Supplementary Methods.

## Results

### GCase Activity Decreased in PD Brains

**FIGURE 1:**
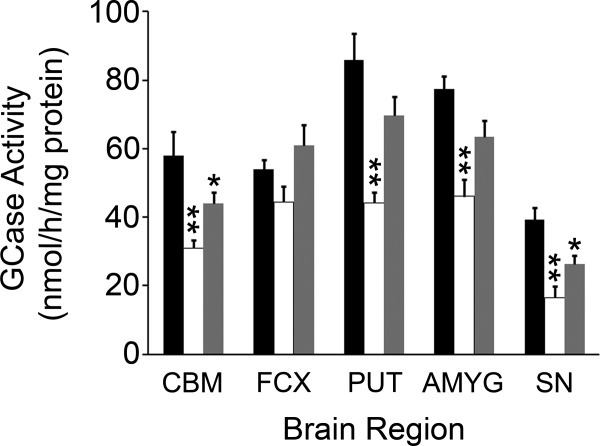
Glucocerebrosidase enzyme (GCase) deficiency in Parkinson disease (PD) brains carrying *GBA* mutations and sporadic PD. GCase activity was significantly decreased in the cerebellum (CBM, n = 14), putamen (PUT, n = 12), amygdala (AMYG, n = 12), and SN (SN, n = 9), but not the frontal cortex (FCX, n = 14) of PD brains with *GBA* mutations (PD+GBA, *white bars*), when compared to controls (n = 6–10, *black bars*). GCase activity was also significantly decreased in the CBM (n = 14) and SN (n = 14) of sporadic PD brains *(gray bars)*, but not the FCX (n = 14), PUT (n = 14), or AMYG (n = 12). *p < 0.05 versus control, **p < 0.01 versus control.

GCase activity was assayed in the cerebellum, frontal cortex, amygdala, putamen, and SN of control brains, PD brains from *GBA* mutation carriers (PD+GBA), and sporadic PD brains (Fig 1). GCase activity was significantly decreased (*p* < 0.01) in all PD+GBA brain regions except the frontal cortex, when compared to control. GCase deficiency was greatest in the SN (58% decrease), whereas GCase activity was decreased in the cerebellum (47%), frontal cortex (17%), amygdala (40%), and putamen (48%). GCase activity was also significantly decreased (*p* < 0.05) in the cerebellum by 24% and SN by 33% in sporadic PD brains.

GCase activity was unaffected in the amygdala of Alzheimer disease patients, suggesting that the deficiency seen in the SN of sporadic PD brains was not simply a consequence of neurodegeneration (Supplementary Fig 2).

The activity of nonlysosomal GCase (GBA2)^21^ was not significantly affected in any region of either PD+GBA or sporadic PD brains (Supplementary Fig 3).

### GCase Protein Levels Decreased in PD Brains

**FIGURE 2:**
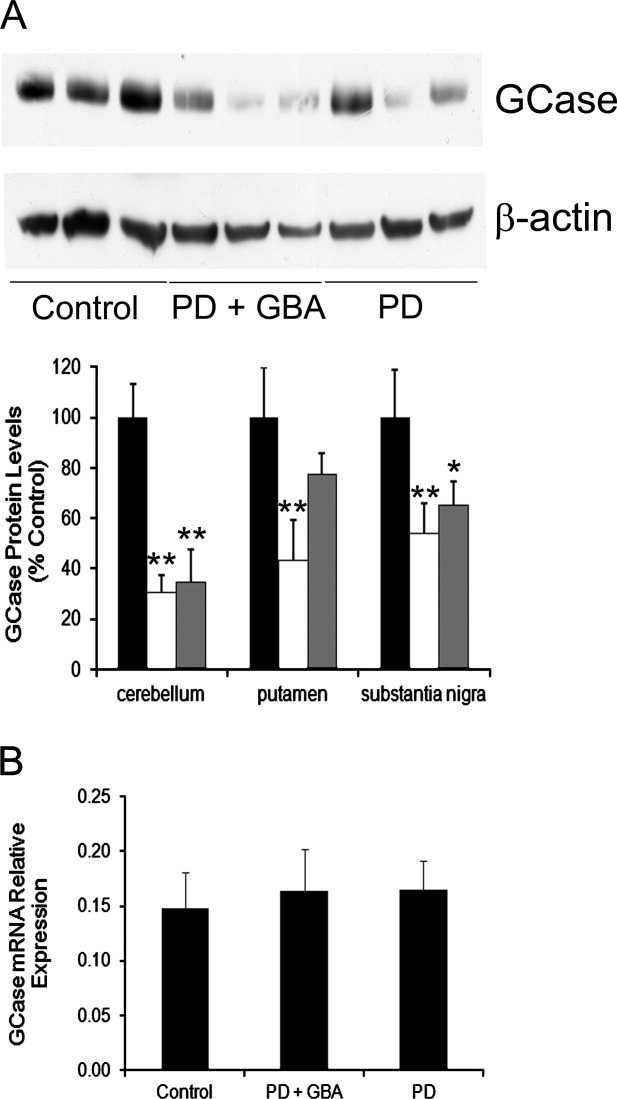
Glucocerebrosidase enzyme (GCase) protein expression was decreased in Parkinson disease (PD) brains carrying *GBA* mutations and sporadic PD brains. (A) Western blotting for GCase protein levels in cerebellum, putamen, and substantia nigra (example blot shown). GCase protein was significantly decreased in the cerebellum of both PD+GBA *(white bars)* and sporadic PD brains (*gray bars*, PD+GBA, 30.4 ± 7.0%, n = 14; PD, 34.6 ± 13.1%, n = 6, of control brain optical density *[black bars]*, 100 ± 13%, n = 9). GCase was significantly decreased in the putamen of PD+GBA brains but not sporadic PD brains (PD+GBA, 43.4 ± 16.0%, n = 7; PD, 77.2 ± 8.8%, n = 6, of control brain optical density, 100 ± 20%, n = 7). GCase was significantly decreased in the substantia nigra of PD+GBA brains and sporadic PD brains (PD+GBA, 53.9 ± 12.0%, n = 9; PD, 65.1 ± 9.3%, n = 11, of control brain optical density, 100 ± 19%, n = 7). (B) RNA was extracted from the putamen of control (n = 6), PD+GBA (n = 10), and sporadic PD brains (n = 6), and GCase mRNA relative expression was measured with quantitative real time polymerase chain reaction. No changes in mRNA were detected. *p < 0.05 versus control, **p < 0.01 versus control.

GCase protein levels were measured in PD+GBA and sporadic PD brains to determine whether the deficiency in GCase activity was due to inhibition of the enzyme or decreased protein levels. GCase protein levels were significantly decreased in the cerebellum (*p* < 0.01), putamen (*p* < 0.01), and SN (*p* < 0.01) of PD+GBA brains (Fig 2A). In PD brains, GCase levels were significantly decreased in the cerebellum (*p* < 0.01) and SN (*p* < 0.05). Similar to a previous report on GCase expression in human brain,^18^ only 1 predominant GCase species (∼60kDa) was detected. Two GCase species were seen in the neuroblastoma cell line SH-SY5Y, the lower of which migrates exactly the same way as GCase detected in brain (Supplementary Fig 4). Silencing of GCase in SH-SY5Y cells with small interfering RNA (siRNA) significantly diminished both GCase species, confirming the veracity of the antibody.

To eliminate the possibility that the decreased GCase protein levels in Triton X-100–soluble brain homogenates was due to the protein becoming insoluble, brains were sequentially homogenized in high salt, high salt + Triton X-100, and urea + sodium dodecyl sulfate (insoluble fraction). Western blotting of soluble and insoluble fractions of brain samples showed that GCase was predominantly located in Triton X-100–soluble fractions in all 3 groups, and that the decreased GCase protein levels in PD+GBA and PD brains was not due to a greater proportion of GCase becoming Triton X-100 insoluble (see Supplementary Fig 4). There was a trend for increased levels of monomeric α-synuclein in PD+GBA and PD brains, with less α-synuclein being soluble in the high-salt fraction.

GCase mRNA levels were unaffected in the putamen of PD+GBA and sporadic PD brains (see Fig 2B), indicating that the decreased protein levels were not due to reduced transcription of the *GBA* gene. There was not enough material for RNA analysis of the SN.

**FIGURE 3:**
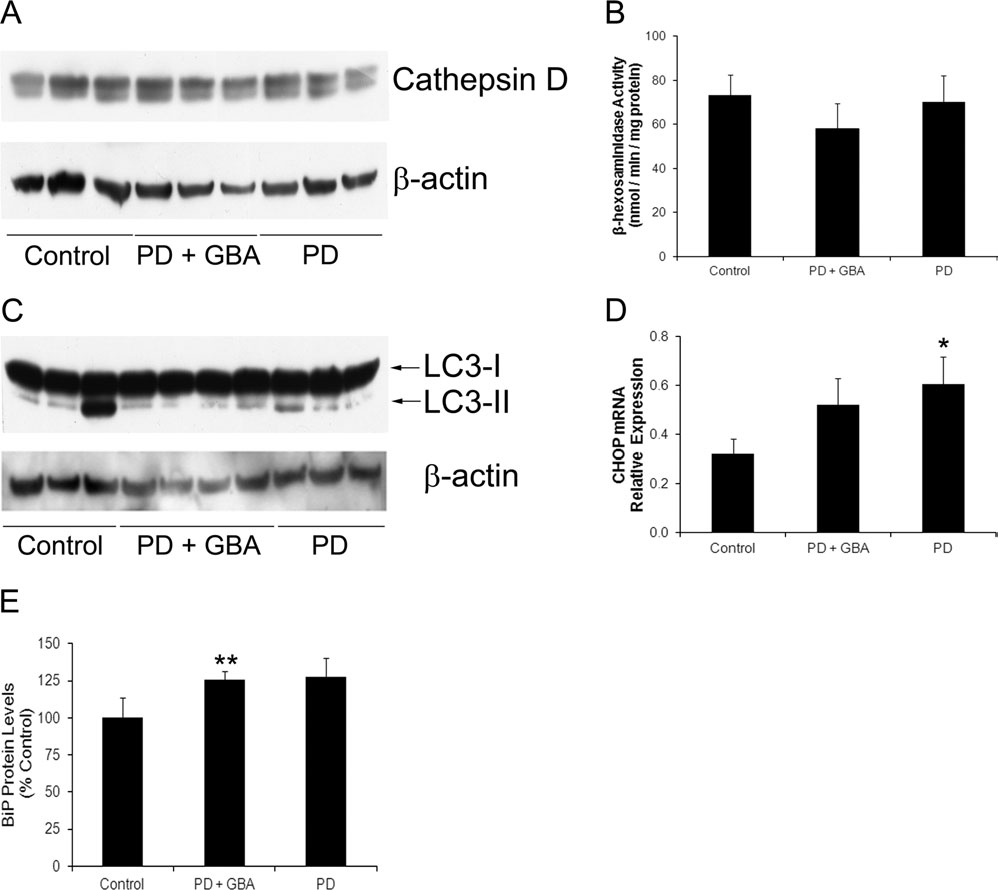
Glucocerebrosidase enzyme deficiency was not due to decreased lysosomal content, but C/EBP homologous protein (CHOP) and binding immunoglobulin protein (BiP) levels were increased. (A) Western blots for cathepsin D in the substantia nigra (SN) of control brains, PD brains carrying *GBA* mutations (PD+GBA), and sporadic PD brains (PD) was unaffected. (B) The activity of the lysosomal enzyme β-hexosaminidase in the SN of control (n = 7), PD+GBA (n = 9), and PD (n = 14) was unaffected. (C) Measurement of LC3-II levels in the putamen did not differ significantly between the groups (control, 0.624 ± 0.329, n = 6; PD+GBA, 1.122 ± 0.596, n=7; PD, 0.816 ± 0.213, n=6). (D) CHOP mRNA levels were increased in the putamen of both PD+GBA (n = 10) and PD brains (n = 6) compared to control brain (n = 6). (E) Protein levels of the chaperone BiP were found to be increased by western blotting in the putamen of both PD+GBA (n = 8) and PD brains (n = 10) compared to control brain (n = 8). *p < 0.05 versus control, **p < 0.01 versus control.

The decrease in GCase protein expression was not associated with a decrease in lysosomal content. Expression of the lysosomal enzyme cathepsin D was unaffected in cerebellum, putamen, and SN (shown) in PD+GBA and sporadic PD brains (Fig 3A), and β-hexosaminidase activity in the SN was similar in all groups (see Fig 3B).

LC3-II protein levels can be used as a measure of autophagosomal number.^23^ Autophagosomes are double membrane structures that carry macromolecules and organelles destined for degradation to lysosomes. There was a trend for higher LC3-II in GBA+PD and sporadic PD putamen, although this was not significant (see Fig 3C).

Certain GCase mutants (eg, N370S, L444P) have been reported to be retained in the endoplasmic reticulum (ER), triggering the unfolded protein response (UPR) and undergoing ER-associated degradation (ERAD) via the proteasome.^24^–^26^ We measured 3 markers of the UPR: splicing of the transcription factor Xbp1, increased transcription of C/EBP homologous protein (CHOP), and increased protein levels of the chaperone binding immunoglobulin protein (BiP).^25^ CHOP mRNA levels were increased in the putamen of PD+GBA (163%) and sporadic PD brains (189%; *p* < 0.05; see Fig 3D). BiP protein levels were increased in the putamen of PD+GBA (126%; *p* < 0.01) and sporadic PD brains (128%; see Fig 3E). Splicing of Xbp1 was detected in 3 of 10 PD+GBA brains (N370S/wt, R463C/wt, L444P/wt) and 2 of 6 sporadic PD brains, compared to 1 of 6 control brains (Supplementary Fig 5).

### Increased α-Synuclein Levels Cause GCase Deficiency

**FIGURE 4:**
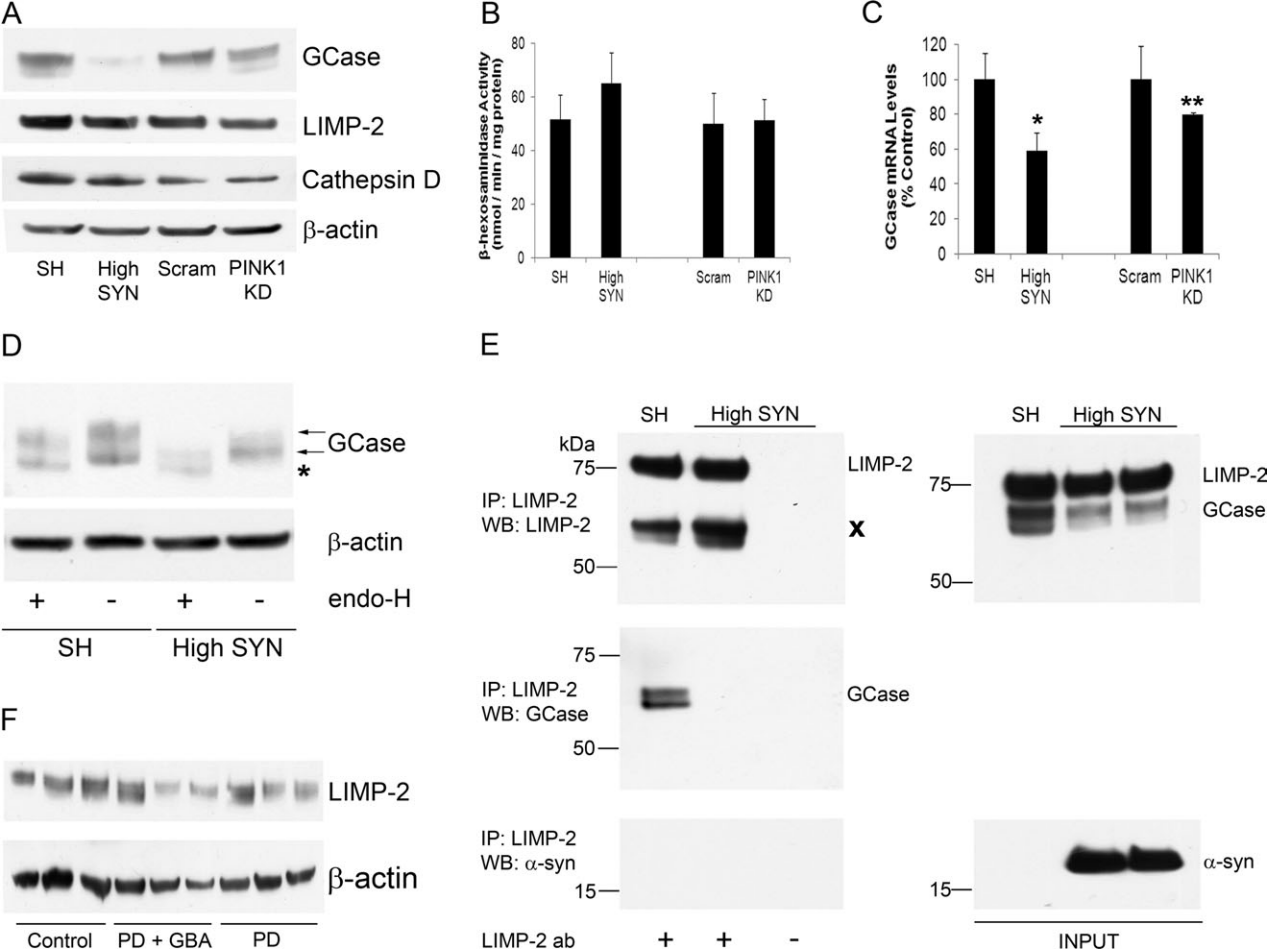
Glucocerebrosidase enzyme (GCase) protein levels were decreased in SH-SY5Y cells overexpressing α-synuclein (α-syn) or with PTEN-induced putative kinase 1 (PINK1) deficiency. (A) Western blotting for GCase in α-synuclein overexpressing cells (High SYN) or PINK1 knockdown (KD) cells showed a significant decrease in protein levels (High SYN, 13.4 ± 7.1%, n = 3, *p* < 0.001; PINK1 KD, 65.3 ± 9.3%, n = 3, *p* < 0.05, of respective control optical density [SH-SY5Y cells, 100 ± 16%, n = 3; scram short hairpin RNA cells, 100 ± 9%, n = 3]). Cathepsin D and lysosomal integral membrane protein (LIMP)-2 protein levels were unaffected in either cell type, compared to their respective controls. (B) The activity of the lysosomal enzyme β-hexosaminidase was unaffected in High SYN or PINK1 KD cells (n = 6), when compared to respective control cell lines. (C) Quantitative real time polymerase chain reaction indicated that steady-state GCase mRNA levels were decreased in cells with exogenous α-synuclein or PINK1 KD (High SYN, 58.9 ± 10.1%, n = 3; PINK1 KD, 79.9 ± 1.3%, n = 3 of respective control GCase mRNA levels, n = 3). (D) SH or High SYN cell lysates (20μg) were treated with or without endoglycosidase-H (endo-H) and GCase protein species analyzed by Western blot. The 2 normal species of GCase detected in SH cells are indicated by arrows. An additional lower molecular weight band was observed in High SYN but not SH cells following endo-H treatment *(asterisk)*. (E) LIMP-2 was immunoprecipitated (Ip) from lysates of SH or High SYN cells to a similar extent *(left panel)*. No LIMP-2 was immunoprecipitated from lysates incubated without antibody (ab). Cross denotes heavy chain of antibody used for immunoprecipitation. Two bands corresponding to GCase were pulled down in SH cells, but not High SYN cells *(left panel)*. α-Synuclein was not pulled down in SH or High SYN cells *(left panel)*. The right panel shows the expression of GCase, LIMP-2, and α-synuclein in the initial lysates (INPUT). WB, western blotting (F) Western blotting for LIMP2 in the substantia nigra from control, PD+GBA, or sporadic PD brains showed that there was no difference in expression between the groups (PD+GBA, 92.0 ± 17.2%, n = 8; PD, 123.3 ± 10.7%, n = 8, of control optical density, n = 7). *p < 0.05 versus SH, **p < 0.01 versus scram.

Because GCase protein expression was decreased in sporadic PD brains, we investigated whether increased α-synuclein levels affected GCase levels in the human neuroblastoma SH-SY5Y cell line. Two SH-SY5Y stable cell lines expressing either high levels of exogenous wild-type α-synuclein protein (High SYN) or approximately 10-fold lower levels of exogenous α-synuclein (SYN) were used. (Supplementary Fig 6; normal SH-SY5Y cells contain endogenous α-synuclein, but it is undetectable by western blot.^10^ Exogenous α-synuclein has a hemagglutinin tag at the C-terminal.) GCase enzyme activity was significantly decreased by 70% in High SYN cells (SH cells, 145.7 ± 26.0nmol/h/mg protein; High SYN, 44.1 ± 6.8nmol/h/mg, n = 3; *p* < 0.05), whereas GCase protein levels were significantly decreased by 87% (Fig 4; n = 3; *p* < 0.001). GCase protein levels were decreased by 33% in SYN cells (see Supplementary Fig 6). Protein levels of cathepsin D and the activity of β-hexosaminidase activity were unaffected by increased α-synuclein. It should be noted that steady-state GCase mRNA levels in High SYN cells were decreased by 41% (*p* < 0.05).

Increased α-synuclein levels have been reported to inhibit intracellular trafficking of GCase.^18^ The sensitivity of GCase to endo-H digestion can indicate that GCase is becoming trapped prior to reaching the mid-Golgi apparatus.^24^ Mature, properly folded glycoproteins that have reached the mid-Golgi apparatus are not substrates of endo-H. An additional GCase band of lower molecular weight was detected in High SYN cells after endo-H treatment that was not present in SH cells, indicating that a proportion of GCase was becoming trapped in cells with increased α-synuclein levels (see Fig 4D).

### Loss of Mitochondrial Function Causes GCase Deficiency

Mitochondrial dysfunction and oxidative stress have been implicated in PD pathogenesis.^2^ Treatment of cells with the complex I inhibitor rotenone, the glutathione synthesis inhibitor L-BSO, or the reactive oxygen species generator paraquat for 48 hours had no affect on GCase protein levels (Supplementary Fig 7).

The half-life of GCase has been reported to be 30 hours in cultured cells, with de novo GCase synthesis continually replacing degraded protein.^27^ The toxic nature of the above treatments meant that cells were only treated for 48 hours. Therefore, we investigated GCase expression in SH-SY5Y cells with constitutive PTEN-induced putative kinase 1 (PINK1) knockdown (KD) by short hairpin RNA (shRNA). Mutations in *PINK1(PARK6)* are a cause of autosomal recessive PD. Loss of the mitochondrial kinase PINK1 results in mitochondrial dysfunction and increased oxidative stress.^28^, ^29^ In PINK1 RNAi cells, GCase activity was decreased by 30% (scrambled control, 257.0 ± 35.0nmol/h/mg protein; PINK1 KD, 180.3 ± 45.1nmol/h/mg protein; n = 3; *p* < 0.05). Steady-state GCase protein and mRNA levels were decreased by 35% and 20%, respectively (n = 3; *p* < 0.01), when compared to control (scram) cells (see Fig 4A, C). GCase activity was significantly decreased by 24% (n = 4; *p* < 0.05) in a different RNAi clone. Transient transfection of a PINK1 siRNA cocktail into SH-SY5Y cells also decreased GCase protein levels and activity, which could be rescued by exogenous expression of PINK1 (Supplementary Fig 8).

### GCase Transporter LIMP-2 Was Unaffected in PD Brains or SH-SY5Y Cells with Increased α-Synuclein Levels

Unlike other lysosomal hydrolases, GCase is targeted to lysosomes independently of the mannose-6-phosphate receptor.^30^ GCase specifically binds the lysosomal integral membrane protein LIMP-2 in the ER and is transported via the Golgi apparatus to lysosomes. Western blotting for LIMP-2 in High SYN cells indicated that LIMP-2 levels were unaffected (see Fig 4A). To determine whether α-synuclein binds to LIMP-2 and interferes with GCase trafficking, LIMP-2 was immunoprecipitated in control and High SYN cells. LIMP-2 failed to pull down α-synuclein (see Fig 4E). Two bands corresponding to GCase were pulled down in control cells but were absent in α-synuclein overexpressing cells. These data suggest that increased α-synuclein levels do result in decreased amounts of GCase being delivered to lysosomes by LIMP-2. However, this does not appear to be due to a direct interaction between LIMP-2 and α-synuclein.

Western blotting for LIMP-2 in the SN of PD+GBA brains or sporadic PD brains (see Fig 4F) indicated that LIMP-2 protein levels were unaffected.

## Discussion

We report the first comprehensive biochemical analysis of the effects of *GBA* mutations in PD brains. There is widespread deficiency of GCase activity and protein levels in PD+GBA brains, with the most severe defect located in the SN (58%) and putamen (48%). This loss of GCase is unlikely to simply represent neurodegeneration, as there was a 47% defect in cerebellum, an area not involved in the degenerative process of PD. GCase activity was also unaffected in the amygdala of AD brains, a region associated with marked neurodegeneration. There was no decrease in GCase mRNA levels in the putamen, indicating that the reduction in protein levels, in this region at least, was not a result of downregulation of gene expression, but rather a post-translational effect on protein levels. Three markers of UPR/ERAD were increased and might play a role in reduced protein levels, although further studies are required to confirm this. Given the normal cathepsin D levels and hexosaminidase activity, the loss of GCase protein was not due to a general reduction in lysosomal content or activity. There was a trend for LC3-II levels to be increased in PD+GBA putamen, suggesting an increase in autophagosome number, a feature previously reported in PD SN and amygdala.^10^, ^11^ As autophagy flux cannot be measured in postmortem tissue, it is unclear whether this is due to an increase in macroautophagy or an inhibition of lysosomal degradation of autophagosomes.

We also report for the first time a significant deficiency of GCase activity in PD SN (33%) and cerebellum (24%), with reduced protein levels, in sporadic PD brains. Enzyme activities were comparable to control in other PD brain regions. The patterns of lysosomal proteins and UPR/ERAD markers were similar in PD and GBA+PD brains.

The SN is the site of greatest pathology in PD brain and biochemical abnormalities thought relevant to PD pathogenesis, including α-synuclein deposition, mitochondrial dysfunction, and oxidative stress. Although the loss of GCase activity in PD+GBA brains is in part related to *GBA* mutations, this cannot be the explanation in PD brains. Thus, we hypothesized that mitochondrial dysfunction and/or oxidative stress, or increased α-synuclein levels might cause this defect in PD and an exacerbation of the GCase deficiency in PD+GBA. Mitochondrial dysfunction/oxidative stress as a result of PINK1 KD^28^, ^29^ caused a loss of GCase activity and protein in cells. Increased α-synuclein expression also significantly decreased GCase activity and protein levels.

Recent studies have highlighted the reciprocal relation between GCase activity and α-synuclein. Decreased GCase activity caused increased α-synuclein levels in toxin and transgenic GBA mouse models, and in neuronal cultures.^14^–^18^ Accumulation of the GCase substrate glucosylceramide can stabilize soluble α-synuclein oligomers in vitro,^18^ and oligomeric α-synuclein has been reported in patients with homozygous or heterozygous *GBA* mutations with Lewy body dementia, although not in PD.^18^, ^31^ No statistical differences in Lewy body pathology were reported between sporadic PD brains and PD+GBA brains studied here.^8^ We also found no noticeable difference in the solubility or amount of monomeric α-synuclein between PD+GBA brains and sporadic PD. Differences in α-synuclein conformation between these groups warrants further investigation.

GCase has also been shown to bind α-synuclein at lysosomal pH, and increased α-synuclein levels in the cortex can result in the depletion of lysosomal GCase.^18^, ^19^ Our in vitro data suggest that increased sensitivity of GCase to endo-H in cells with increased α-synuclein results in the aberrant trafficking of GCase through the ER. α-Synuclein can affect ER/Golgi apparatus trafficking,^32^, ^33^ but the mechanism by which GCase transport is reduced remains unclear. LIMP-2, the protein required for GCase transport to the lysosome,^30^ does not bind α-synuclein. However, less GCase is bound to LIMP-2 in α-synuclein–overexpressing cells, and therefore the amount of enzyme delivered to the lysosome is decreased. LIMP-2 levels were unaffected in the SN of PD brains. The decrease in steady-state GCase mRNA levels observed in vitro needs further investigation to determine whether this is a decrease in transcription or increased degradation of mRNA, and the extent to which this contributes to GCase deficiency.

Accumulation of proteins in the ER can induce UPR/ERAD. The 2 most common heterozygote *GBA* mutations associated with PD (N370S and L444P)^7^ have been reported to undergo UPR/ERAD in cultured cells.^24^, ^25^, ^34^ However, 2 Gaucher mouse studies could not find evidence of UPR/ERAD in the brain.^17^, ^35^ UPR/ERAD markers were increased in both PD brains with *GBA* mutations and sporadic PD. Aberrant trafficking of GCase might contribute to this increase in UPR/ERAD. However, it could also be a result of perturbed calcium homeostasis, redox status, or proteostasis or mitochondrial dysfunction, all of which are linked with PD pathogenesis.^26^, ^36^

Surprisingly, GCase activity was decreased in the cerebellum of PD and PD+GBA brains, although this is not a site associated with neurodegeneration, α-synuclein accumulation,^37^ mitochondrial dysfunction, or oxidative stress in PD.^38^ It is unclear why GCase activity is decreased in this region. We speculate that deficiency in the cerebellum is caused by a mechanism separate from that occurring in recognized pathogenic areas of PD such as the SN, putamen, and amygdala.

**FIGURE 5:**
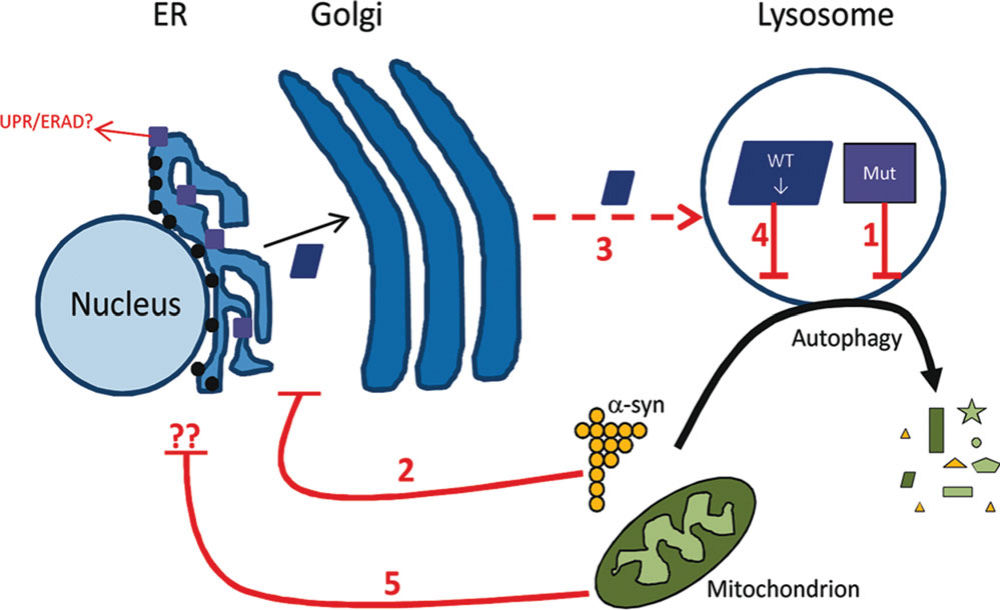
Scheme of glucocerebrosidase enzyme (GCase) deficiency in PD pathogenesis. Mutant (Mut) GCase *(purple squares)* disrupts the degradation of α-synuclein and organelles such as mitochondria by the autophagy–lysosomal pathway (1). Some GCase mutants may also become trapped in the endoplasmic reticulum (ER) and activate the unfolded protein response (UPR)/endoplasmic reticulum-associated degradation (ERAD). Increased levels of α-synuclein (α-syn) impair the trafficking of wild-type (WT) GCase *(blue rectangle)* via the ER/Golgi apparatus (2), resulting in less WT GCase being delivered to the lysosomes by lysosomal integral membrane protein-2 (3). This will exacerbate any lysosomal/autophagic dysfunction (4). Dysfunctional mitochondria can also affect WT GCase protein levels by an unidentified mechanism (5).

In conclusion, our studies confirm a widespread deficiency of GCase activity in the brains of PD patients carrying *GBA* mutations. We also demonstrate that PD patients without *GBA* mutations exhibit deficiency of GCase in SN. Based upon the relation between GCase, α-synuclein, and mitochondrial function shown here and by others, we propose that PD pathology is exacerbated and accelerated, but not necessarily initiated, by *GBA* mutations (Fig 5). This would explain why not all *GBA* mutation carriers develop PD, and why those who do tend to do so at an earlier age.
