# Supplementary material for: Glucocerebrosidase Deficiency in Substantia Nigra of Parkinson Disease Brains
Source: Ann Neurol. 2012 Oct 2;72(3):455–63. doi: 10.1002/ana.23614 (PMC3638323; doi:10.1002/ana.23614)
Supplement: Supplementary file 1 [file ana0072-0455-SD1.doc]

**Supplementary Methods**

Unless stated all chemicals were purchased from Sigma-Aldrich.

**Tissue Culture**

SH-SY5Y cells were cultured in 1:1 DMEM/F12 (Invitrogen) supplemented with 10% FCS, non essential amino acids, 1 mM sodium pyruvate and penicillin-streptomycin. Following treatment with toxins, cells were harvested by trypsinisation and lysed on ice in 0.5% (v/v) Triton X-100 in PBS supplemented with protease inhibitors for 15 minutes. Nuclei and debris were removed by centrifugation at 17 000 x g for 10 minutes, 4 °C. Protein concentration was measured using BCA kit (Pierce).

PINK1 RNAi cells were generated by transfecting SH-SY5Y cells with HuSH shRNA plasmid (Origene) containing 29mer shRNA against PINK1 (5’-GCTGTGTATGAAGCCACCATGCCTACATT) or scrambled shRNA and positive clones selected using puromycin. Cells were cultured as above. Quantification of PINK1 mRNA indicated that PINK1 was silenced by 78%, when compared to clones expressing scrambled shRNA.

SH-SY5Y cells contain endogenous α-synuclein but it is undetectable by western blot (Alvarez-Erviti et al., 2010). To investigate the effect of increased α-synuclein on GCase, two SH-SY5Y stable cell lines expressing high (High SYN) or low (SYN) levels of exogenous wild-type α-synuclein were used. Exogenous α-synuclein protein levels were approximately 10-fold higher in High Syn, when compared to SYN cells (Supplementary Figure 6; Alvarez-Erviti et al., 2010). α-synuclein was cloned in to pcDNA3.1, transfected in to SH-SY5Y cells, and positive clones selected using the antibiotic G418 (20 μg/ml). Exogenously expressed α-synuclein contains a hemagglutinin tag (HA) at the C-terminal.

Stable-transfected SH-SY5Y cells with wild-type PINK1 have previously been described (Gegg et al., 2009).

**Transient transfection of siRNA**

SH-SY5Y cells (1.8 x 10^5^ cells/ml) were transfected with 25 nM GBA siRNA (Dharmacon; sense: GGAUGUGCCUCUUACCAUCUU; antisense: GAUGGUAAGAGGCACAUCCUU) or 25 nM scrambled control #1 (Ambion) for 3 days. SH-SY5Y were then passaged and transiently transfected as above with siRNA. This was repeated on day 6 and cells harvested by trypsinisation and lysed as above on day 9.

SH-SY5Y cells or PINK OE cells were transfected with a cocktail of two different PINK1 siRNA (10 nM total) for 6 days as described previously (Gegg et al., 2009). Endogenous PINK1 mRNA levels were decreased by 85% in SH-SY5Y cells. Endogenous PINK1 protein cannot be detected by western blotting. Exogenous PINK1 expression was detected, even after PINK1 siRNA treatment (Gegg et al., 2009).

**Fractionation of brain tissue**

Analysis of GCase and α-synuclein in amygdala brain samples was performed as described by Waxmann & Giasson (2008). Tissue (20 mg) was homogenized in 3 ml/mg of high-salt (HS) buffer (50 mM Tris, pH 7.5, 750 mM NaCl, 5 mM EDTA, supplemented with protease inhibitors (1 mM of phenylmethylsulfonyl,1 mg/ml each of pepstatin, leupeptin) and 1 mM NaVO_4_ phosphatase inhibitor. Samples were centrifuged at 20,000 × *g* for 10 minutes, and supernatants kept for analysis. Pellets were then homogenized in HS containing 1% (v/v) Triton X-100 (TX), 6 units DNase, and protease/phosphatase inhibitors, centrifuged as above, and supernatant kept for analysis. The pellet from the TX solubilisation step was then homogenised in urea/SDS (US; 8 M urea, 2% (w/v) SDS, 10 mM Tris, pH 7.5, 6 units DNase, and protease/phosphatase inhibitors), centrifuged, and supernatant kept for analysis. Protein concentration from the HS, TX and US supernatants were then measured by a BCA kit and 30 μg protein from each fraction heated at 70 °C for 10 minutes (except US samples), loaded on to 4-12% Bis-Tris NuPAGE denaturing gels and western blots for GCase and α-synuclein performed. Protein levels were normalised against the density of β-actin band in each fraction. The total sum of GCase/ α-synuclein in HS, TX and US fractions was calculated, and the percentage of the respective proteins in the three fractions calculated.

**Endoglycosidase H Assay**

Endoglycosidase H assay was performed as per instructions (New England Biolabs). Cells were lysed in 0.5% (v/v) TX-100 in PBS on ice for 15 minutes and insoluble material/debris removed by centrifugation (17 000 x g, 4 °C for 10 minutes). Protein (20 μg) was denatured with 1X glycoprotein denaturing buffer at 100 °C for 10 minutes. Protein was then incubated with 1000 units of endoglycosidase H in 1X reaction buffer for 1 hour at 37 °C. Reaction was stopped by the addition of NuPAGE gel loading buffer (Invitrogen) and heated at 70 °C for 10 minutes. GCase was then detected by western blotting.

**Quantitative PCR**

RNA was extracted from tissue (≤ 15 mg) or cultured cells (< 10^6^ cells) using the RNeasy kit (Qiagen) and converted to cDNA with QuantiTect reverse transcription kit (Qiagen). Relative expression of CHOP, GCase, and β-actin mRNA was measured with Power SYBRgreen kit (Applied Biosystems) using a STEP One PCR machine (Applied Biosystems). β-actin mRNA levels were used to normalise data. Primers are listed in Supplementary Table 1. Relative expression was calculated using the ΔC_T_ method.

**Xbp-1 Splicing Assay**

RNA was extracted from putamen (≤ 10 mg) using the RNeasy kit (Qiagen) and converted to cDNA with the QuantiTect reverse transcription kit (Qiagen). PCR was performed using cDNA and DNA polymerse (AmpliTaq 360, Applied Biosystems) using the primers listed in Supplementary Table 1. Annealing temperature was 59 °C. PCR product was separated on 2.5% agarose gel. Unspliced Xbp-1 yielded a product of 289 bp, while the spliced version was 263 bp (Mu et al., 2008).

**LIMP-2 immunoprecipitation**

SH-SY5Y cells or High SYN cells were gown in 60 mm plates until 90% confluent. Cells were harvested with trypsin and lysed with buffer (50 mM Tris, pH 7.5, 0.27 M sucrose, 1% (v/v) NP-40, 10 mM β-glycerophosphate, 1 mM EDTA, 1 mM EGTA, 1 mM sodium orthovanadate, 1 mM PMSF) for 15 minutes on ice. Debris was removed by centrifugation at 17, 000 x g, 10 minutes, 4 °C. An aliquot of lysate (INPUT) was reserved for western blotting. Remaining lysate was rotated with LIMP-2 antibody (1/100; abcam, ab16522) overnight at 4 °C. Protein G-sepharose beads (GE Healthcare) were then added and lysates rotated at room temperature for 2 hours. Beads were washed four times with Tris buffered saline, and protein bound to protein G-sepharose beads released by incubation with 0.1 M glycine, pH 2.9 for 5 minutes. Supernatant was then mixed with gel loading buffer and western blotting performed.

**Antibodies**

α-synclein (LB509, Zymed), BiP (ab21685, abcam) cathepsin D (clone CTD-19, abcam), GCase (clone 2E2, Calbiochem), LIMP-2 (ab16522, abcam), LC3B (#2775, Cell Signaling Technology), β-actin (clone ac-15, abcam), HA tag (SAB4300603, Sigma).

**Alzheimer’s disease brain and short post mortem delay PD sample**

Amygdala samples from control and Alzheimer’s disease patients (n=6; age, 87.2 ± 5.3 years; post mortem delay, 3.0 ± 0.7 hours) and a short post mortem delay PD patient (2.5 hours) were obtained from University Hospital of Bellvitge, Barcelona, Spain, and the Navarra Brain Bank, Pamplona, Spain, with the consent of the local ethics committees.

**Statistical Analyses**

Data are expressed as mean ± S.E.M and statistical significance between groups determined by one-way ANOVA followed by the Tukey HSD test or two-tailed t-Test.

**Supplementary References**

Alvarez-Erviti L, Rodriguez-Oroz MC, Cooper JM, Caballero C, Ferrer I, Obeso JA, Schapira AH. (2010) Chaperone-mediated autophagy markers in Parkinson disease brains. Arch. Neurol. 67:1464-72.

Gegg ME, Cooper JM, Schapira AH, Taanman JW. (2009) Silencing of PINK1 expression affects mitochondrial DNA and oxidative phosphorylation in dopaminergic cells. PLoS One. 4:e4756.

Mu TW, Ong DS, Wang YJ, Balch WE, Yates JR 3rd, Segatori L, Kelly JW. (2008) Chemical and biological approaches synergize to ameliorate protein-folding diseases. Cell. 134:769-81.

Waxman EA, Giasson BI. (2008) Specificity and regulation of casein kinase-mediated phosphorylation of alpha-synuclein. J. Neuropathol. Exp. Neurol. 67:402-16.

| **Target** | **Sequence** | **Annealing Temp (°C)** |
| --- | --- | --- |
| β-actin | 5’-TCT ACA ATG AGC TGC GTG TG-3’  5’-GGT GAG GAT CTT CAT GAG GT-3’ | 58 |
| CHOP | 5'-ACC AAG GGA GAA CCA GGA AAC G-3'  5'-TCA CCA TTC GGT CAA TCA GAG C-3' | 58 |
| GCase | 5’-TGC TGC TCT CAA CAT CCT TGC C-3’  5’-TAG GTG CGG ATG GAG AAG TCA A-3’ | 58 |
| PINK1 | 5’-GGA CGC TGT TCC TCG TTA-3’  5’-ATC TGC GAT CAC CAG CCA-3’ | 56 |
| Xbp-1 | 5'-TTA CGA GAG AAA ACT CAT GGC-3'  5'-GGG TCC AAG TTG TCC AGA ATG C-3' | 59 |

**Supplementary Table 1. Primer Sequences**

**
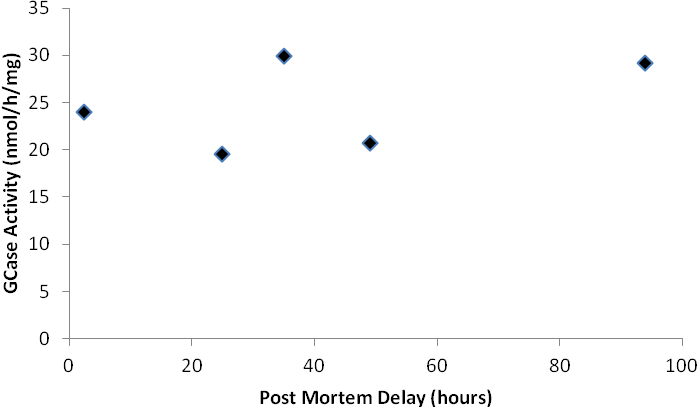
**

**Supplementary Figure 1.** GCase enzyme activity does not diminish with increasing post mortem delay. GCase activity was assayed in the amygdala of PD brains with post mortem delay ranging from 2.5 to 94 hours.


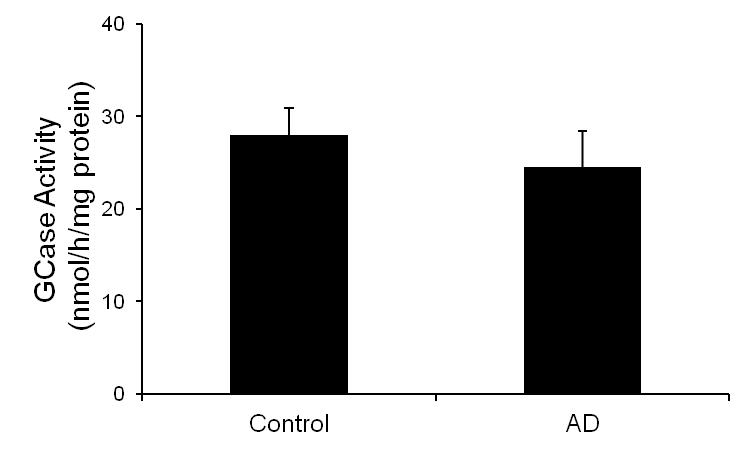


**Supplementary Figure 2:** GCase activity was not significantly decreased in the amygdala of Alzheimer’s disease (AD) brains. GCase activity was assayed in control or AD amygdala (n=6).


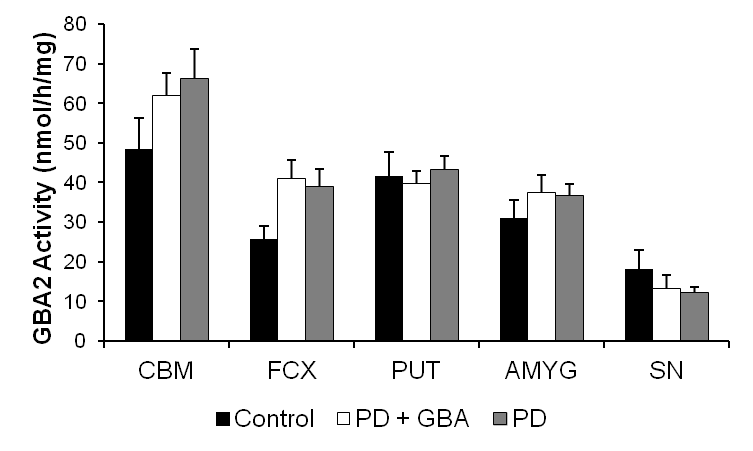


**Supplementary Figure 3:** The activity of non-lysosomal GCase (GBA2) was not significantly affected in any region of either PD+GBA brains (white bars; cerebellum (CBM, n = 14), frontal cortex (FCX, n = 14), putamen (PUT, n = 12), amygdala (AMYG, n = 12) and substantia nigra (SN, n = 9)), or sporadic PD brains (grey bars; CBM , n = 14; FCX, n = 14; PUT, n = 14; AMYG, n = 12: SN, n = 14).

**(A)**

**
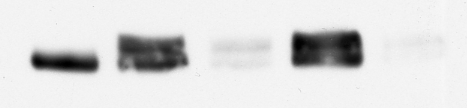
**

50

75

GCase

kDa

**
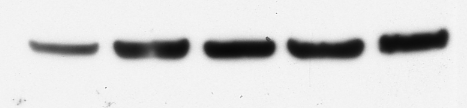
**

Brain scram GCase

siRNA

β-actin

**(B)**

75

**
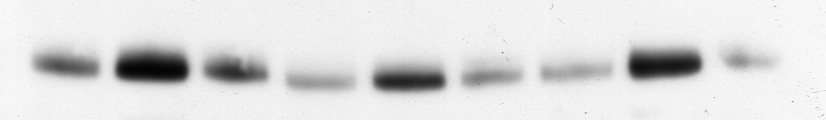
**

GCase

50

**
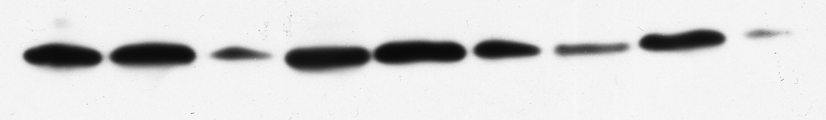
**

15

α-synuclein

**
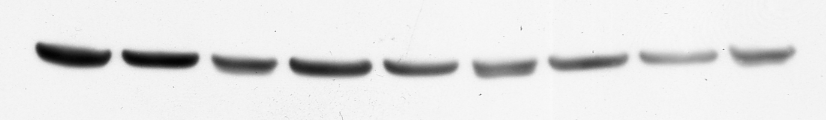
**

β-actin

HS TX US HS TX US HS TX US

control PD + GBA PD

**
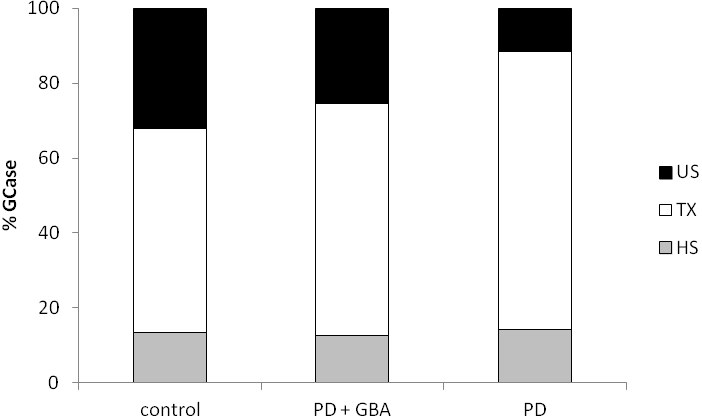

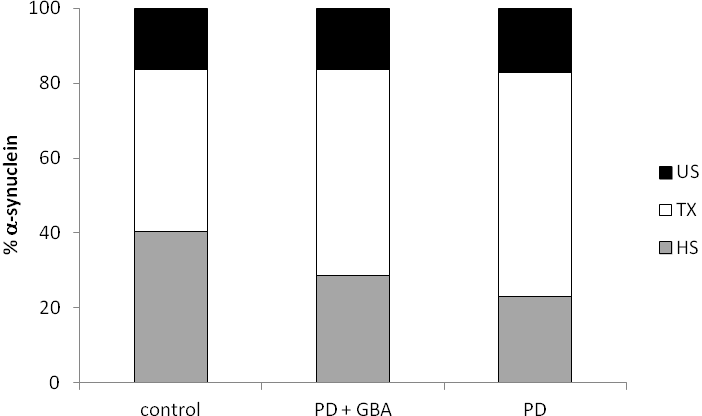
**

**Supplementary Figure 4:** (A) SH-SY5Y cells were transiently transfected with GCase or scrambled siRNA for 9 days. Western blotting for GCase was performed on siRNA lysates and a human brain sample. The GCase band detected in human brain migrated the same as the lower GCase band in SH-SY5Y cells treated with scrambled siRNA. Both GCase bands in SH-SY5Y cells were diminished when cells were treated with GCase siRNA. (B) GCase/α-synuclein was extracted from the amygdala of control, PD+GBA, and PD brains with three sequential buffers: high salt (HS), HS + Triton X-100 (TX), and urea/SDS (US). Protein (30 μg) from each fraction was analysed by western blot. Band density was expressed as a ratio against β-actin. The percentage of GCase/α-synuclein in each fraction was then calculated for the three groups. There was a trend for less α-synuclein in the HS fraction of PD+GBA and PD brains. The sum of α-synuclein in all three fractions of PD+GBA and PD brains was also greater (control, 2.34 ± 0.21; PD+GBA, 3.82 ± 1.04; PD, 3.08 ± 0.69).

500

bp

PD+GBA Con PD PD PD

**
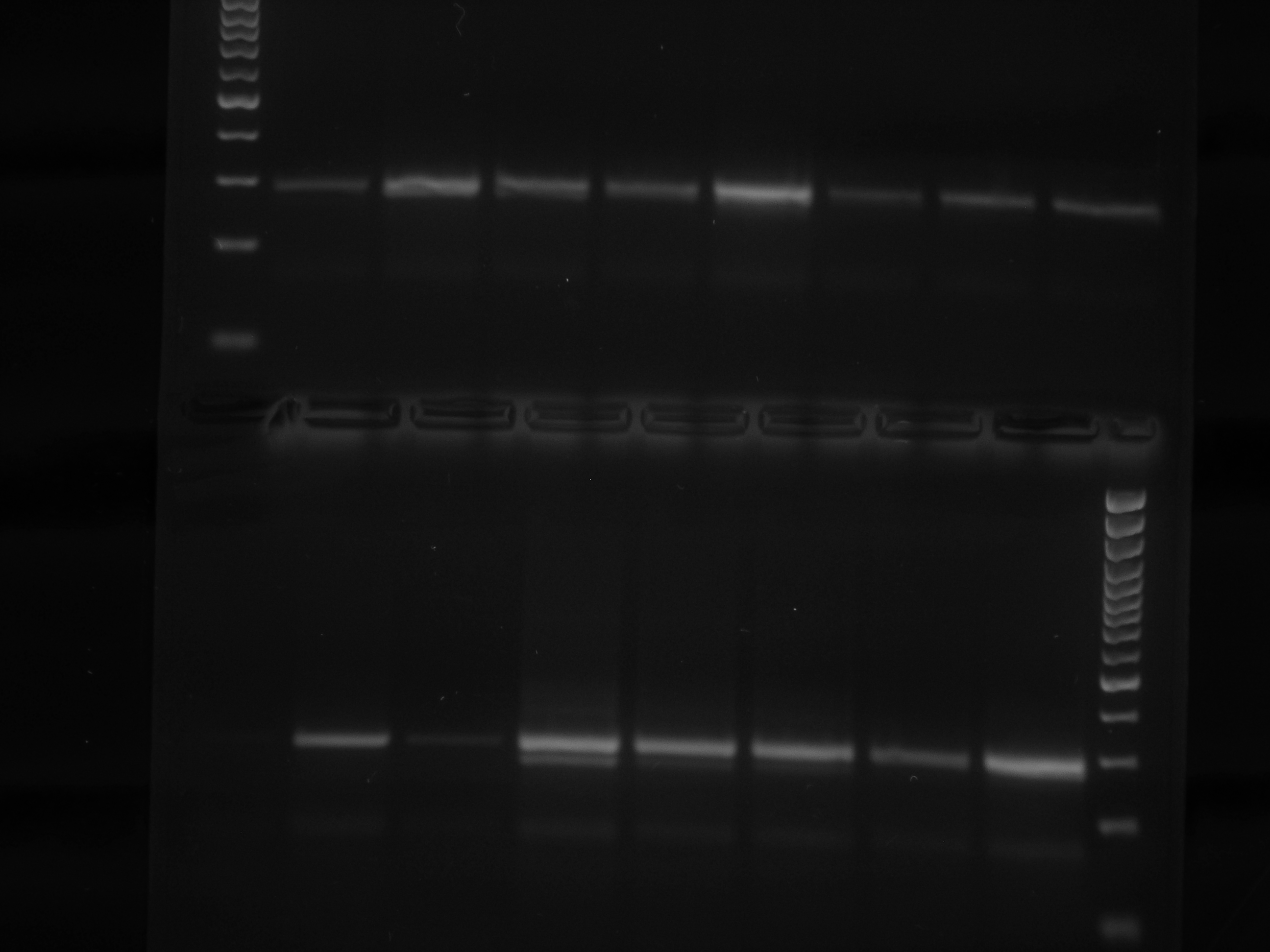
**

400

300

200

100

**Supplementary Figure 5:** Splicing of Xbp-1 in human brain. RNA was extracted from the putamen of control, PD+GBA, or PD brains and splicing of Xpb-1 determined by PCR. Full length Xpb-1 is 289 bp and alternative splicing yields a 263 bp product (denoted by arrow). The PD+GBA sample shown here (lane 1) is from patient # 13 with genotype L444P/wt. The one control patient with Xpb-1 splicing (lane 2) died from an intra-cerebral haemorrhage.

**(A)**

**
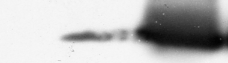
**

HA

**
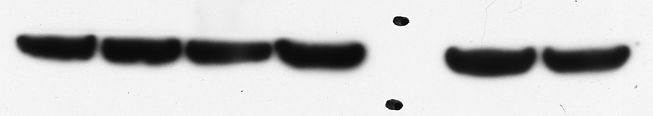
**

β-actin

SH

SYN

High

SYN

**(B)**

**
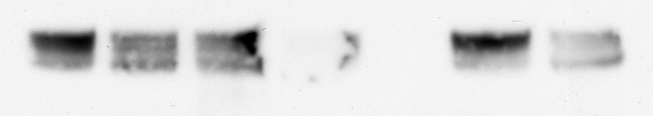
**

GCase

**
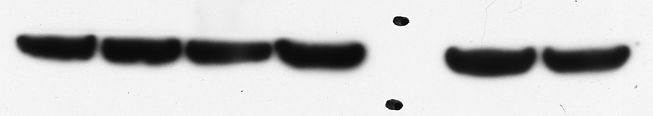
**

β-actin

SH

SYN

**
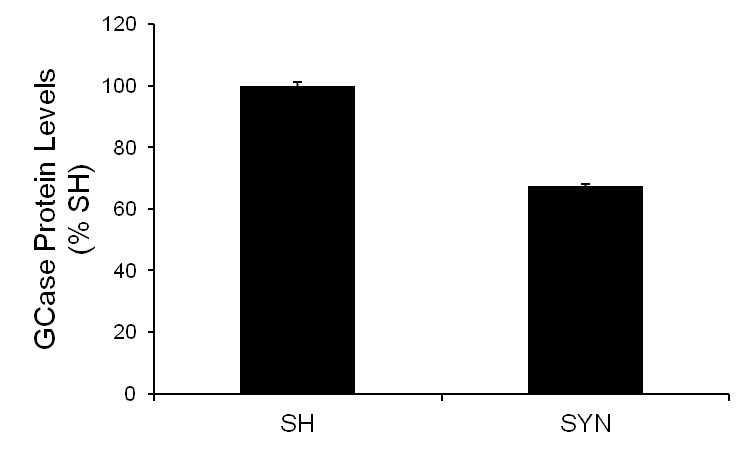
**

******

**Supplementary Figure 6:** GCase protein levels were also decreased in SH-SY5Y cells expressing lower levels of exogenous α-synuclein. (A) Western blot illustrating the expression of exogenous α-synuclein with an HA tag in SH-SY5Y cells. The cell lines expressing lower and higher levels of α-synuclein are denoted SYN and High SYN, respectively. (B) GCase protein levels in SYN cells were decreased by 33% when compared to SH. ***p* < 0.01.

**
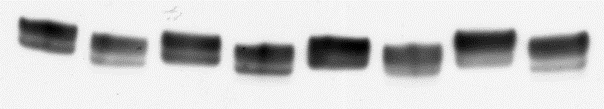

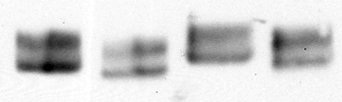
**

GCase

**
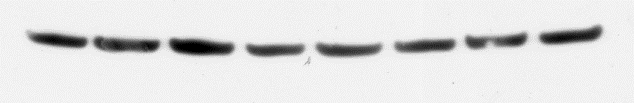

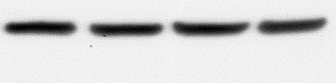
**

**- + - + - + - + - +**

EndoH

β-actin

Control PQ L-BSO Control Rot

**Supplementary Figure 7:** Treatment of SH-SY5Y cells with toxins and their sensitivity to endoglycosidase H. SH-SY5Y cells were treated with 250 μM paraquat (PQ), 1 mM L-BSO or 100 nM rotenone (Rot) for 48 hours. Cell lysates were made and treated with endoglycosidase- H to reveal the proportion of GCase trapped in the ER. Toxin treatment did not result in an increase in lower molecular weight species corresponding to GCase trapped in the ER, when compared to Control.

**(A)**

SH-SY5Y

PINK OE


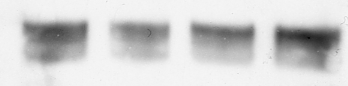


GCase


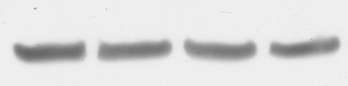


β-actin

Scram PINK1 Scram PINK1

siRNA

*****

**
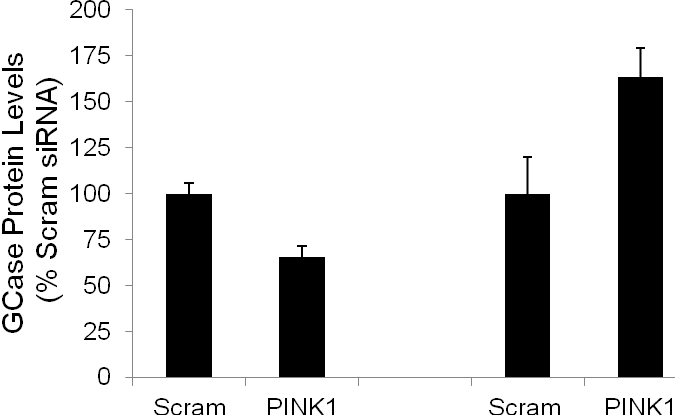
**

******

SH-SY5Y PINK OE

**(B)**


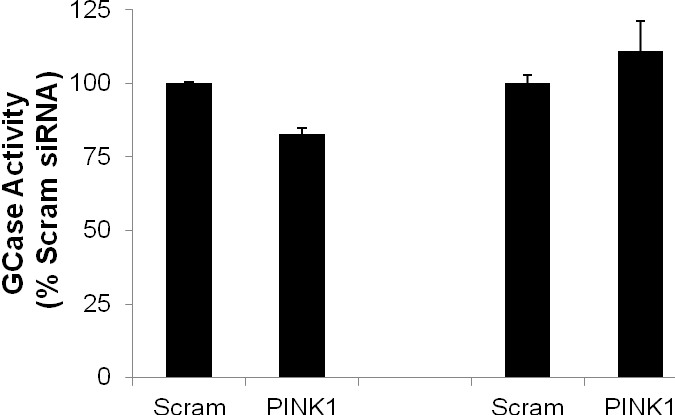


*****

SH-SY5Y PINK OE

**Supplementary Figure 8:** Decreased GCase protein levels and catalytic activity following PINK1-silencing was rescued by expression of exogenous PINK1. (A) SH-SY5Y cells or cells expressing exogenous PINK1 (PINK OE) were treated with scrambled or PINK1 siRNA for 6 days, and GCase protein expression assessed by western blotting. GCase protein levels were decreased in SH-SY5Y cells treated with PINK1 siRNA by 34% (n = 3) when compared to SH-SY5Y treated with scrambled siRNA. GCase protein levels were increased in PINK1 OE cells when treated with PINK1 siRNA (n=3) when compared to PINK-OE cells treated with scrambled siRNA. (B) GCase activity was significantly decreased by 17% (n = 4) in SH-SY5Y cells treated with PINK1 siRNA for 6 days when compared to cells transfected with scrambled siRNA. GCase activity was unaffected in PINK OE cells treated with PINK1 siRNA. * *p* < 0.05 vs. scrambled siRNA; ** *p* < 0.01 vs. scrambled siRNA.
